# Diagnostic test accuracy of ultrasound for orbital cellulitis: A systematic review

**DOI:** 10.1371/journal.pone.0288011

**Published:** 2023-07-06

**Authors:** Mohammed Rashidul Anwar, Sanjay Mahant, Toni Agbaje-Ojo, Quenby Mahood, Cornelia M. Borkhoff, Patricia C. Parkin, Peter J. Gill

**Affiliations:** 1 Child Health Evaluative Sciences, SickKids Research Institute, Toronto, Ontario, Canada; 2 Department of Pediatrics, University of Toronto, Toronto, Ontario, Canada; 3 The Hospital for Sick Children, Toronto, Ontario, Canada; 4 Institute of Health Policy, Management and Evaluation, Dalla Lana School of Public Health, The University of Toronto, Toronto, Ontario, Canada; Shiraz University of Medical Sciences, ISLAMIC REPUBLIC OF IRAN

## Abstract

**Background:**

Periorbital and orbital cellulitis are inflammatory conditions of the eye that can be difficult to distinguish using clinical examination alone. Computer tomography (CT) scans are often used to differentiate these two infections and to evaluate for complications. Orbital ultrasound (US) could be used as a diagnostic tool to supplement or replace CT scans as the main diagnostic modality. No prior systematic review has evaluated the diagnostic test accuracy (DTA) of ultrasound compared to cross-sectional imaging.

**Objective:**

To conduct a systematic review of studies evaluating the DTA of orbital ultrasound compared with cross-sectional imaging, to diagnose orbital cellulitis.

**Methods:**

MEDLINE, EMBASE, CENTRAL, and Web of Science were searched from inception to August 10, 2022. All study types were included that enrolled patients of any age with suspected or diagnosed orbital cellulitis who underwent ultrasound and a diagnostic reference standard (i.e., CT or magnetic resonance imaging [MRI]). Two authors screened titles/abstracts for inclusion, extracted data, and assessed the risk of bias.

**Results:**

Of the 3548 studies identified, 20 were included: 3 cohort studies and 17 case reports/series. None of the cohort studies directly compared the diagnostic accuracy of ultrasound with CT or MRI, and all had high risk of bias. Among the 46 participants, diagnostic findings were interpretable in 18 (39%) cases which reported 100% accuracy. We were unable to calculate sensitivity and specificity due to limited data. In the descriptive analysis of the case reports, ultrasound was able to diagnose orbital cellulitis in most (n = 21/23) cases.

**Conclusion:**

Few studies have evaluated the diagnostic accuracy of orbital ultrasound for orbital cellulitis. The limited evidence based on low quality studies suggests that ultrasound may provide helpful diagnostic information to differentiate orbital inflammation. Future research should focus studies to determine the accuracy of orbital US and potentially reduce unnecessary exposure to radiation.

## Introduction

Periorbital and orbital cellulitis are inflammatory conditions of the eye that can be difficult to distinguish using clinical examination alone, particularly in young children [1]. Clinically, both conditions present with redness and swelling of the eye, but their etiology, management and prognosis are different. In cases of diagnostic uncertainty or to evaluate for orbital cellulitis associated complications (e.g., subperiosteal abscess), cross-sectional imaging is often obtained. Historically, a Computed Tomographic (CT) scan with contrast has been the modality of choice as it is readily available, does not require sedation in young children, provides excellent anatomical differentiation between bones and soft tissue, and can identify inflammation [2]. However, CT scans expose patients to ionizing radiation, which is a concern in pediatric patients due to the association with malignancy [3]. More recently, magnetic resonance imaging (MRI) use has increased, although it appears to be used in addition to CT scans in cases which require repeat diagnostic imaging [4]. In practice, the use of MRI is limited by its availability, cost, repeat imaging after CT [5, 6] and the need for general anesthesia in young children [7].

Ultrasound may offer an alternative imaging modality with the potential to quickly and easily diagnose patients with periorbital versus orbital cellulitis without radiation exposure or need for general anesthesia. Several early studies advocated in favor of ultrasound as a diagnostic tool for ocular inflammation, highlighting its portability and ability to reduce time from presentation to initiation of treatment [8–10]. Despite these promising earlier studies, ultrasound has not become common practice for diagnosis and treatment. Ultrasound as an imaging technology has improved substantially over the last three decades, with recent advances in portability, cost, and access. Physicians are increasingly using bedside ultrasound or point-of-care ultrasound (POCUS) across multiple specialties, including emergency medicine, general practice, pediatrics, and internal medicine [11]. Recent diagnostic test accuracy studies of POCUS for ocular assessments have identified high sensitivity and specificity for certain conditions in adults, such as retinal detachment and vitreous hemorrhage [12, 13].

However, there is limited contemporary research on the diagnostic test accuracy of orbital ultrasound for orbital cellulitis, a clinical area that can be a diagnostic challenge. Given the increasing access and use of ultrasound, it may provide an adjunctive diagnostic tool for clinicians to guide diagnosis and management and could be used for serial assessment to evaluate response. Therefore, our research question/aim was to determine the diagnostic test accuracy of orbital ultrasound in the diagnosis of orbital cellulitis and its complications (e.g., subperiosteal abscess) compared to a diagnostic reference standard (i.e., computed tomography [CT] or magnetic resonance imaging [MRI]) in patients presenting to the emergency department or admitted to hospital with a suspected severe orbital infection.

## Methods

### Search methodology

We performed a systematic review in accordance with the preferred reporting for systematic reviews and meta-analysis (PRISMA-DTA) guidelines. The PRISMA-DTA checklist has been provided as a S1 Appendix) [14]. This review was registered with PROSPERO CRD42021268171; which is available at https://www.crd.york.ac.uk/prospero/display_record.php?ID=CRD42021268171. With the assistance of a health information specialist (QM), we searched CENTRAL, MEDLINE, EMBASE and Web of Science database from inception to August 10, 2022. We also searched ClinicalTrials.gov and the World Health Organization (WHO) International Clinical Trials Portal (ICTRP). The search strategy used MeSH headings and sensitive search terms. The search strategies are outlined in S2 Appendix. Finally, we hand searched reference lists of included articles and reviews for potentially eligible studies that might have been missed in the electronic database searches, conducted snowball searching, and contacted experts in the field.

### Eligibility criteria, data extraction and risk of bias assessment

Patients of any age who were seen at an emergency department (ED) or hospital (any level of care) with suspected orbital infection (i.e., periorbital or orbital cellulitis) were eligible for inclusion. Studies were included if the study participants had undergone an ultrasound (index test) and a diagnostic reference standard (i.e., CT or MRI) within a reasonably close time interval (i.e., defined as within 48 hours). We included any type of ultrasound including those obtained by technologists and point-of-care ultrasounds (POCUS) obtained by healthcare professionals. We included all study types (i.e., case reports, case series, observational studies, randomized controlled trials. There were no restrictions based on country of origin or language of publication. We used Covidence to manage the selected studies [15].

Two review authors (MRA and TAO) independently screened titles and abstracts. The full text of any potentially relevant articles that met the inclusion criteria were then reviewed for eligibility. The review authors (MRA and TAO) independently screened the articles for inclusion and resolved any discrepancies through discussion. When needed, a third review author (PJG) was consulted. The review authors (MRA and TAO) independently extracted data using a custom designed data collection tool (S3 Appendix). Key aspects included in the data extraction form included general characteristics (e.g., year, country of study, time period, study design), population (e.g., age, sex, inclusion/exclusion criteria), imaging modality findings (e.g., signs of inflammation, abscess), timing of diagnostic tests, types of ultrasound probe/transducer, and outcomes (e.g., definitions of orbital cellulitis and complications). The imaging findings indicated the clinical diagnosis as per Chandler’s criteria [16]: I) periorbital (preseptal) cellulitis; II) orbital cellulitis; III) sub-periosteal abscess; IV) orbital abscess; and V) cavernous sinus thrombosis. These distinctions are anatomically based but provide a helpful schematic upon which to evaluate the diagnostic role of ultrasound. We also extracted data on ultrasound techniques that were employed to better visualize the orbit. Potentially relevant studies that were identified but for which the full text could not be obtained despite multiple attempts to contact the authors, were first labelled as “awaiting classification” and subsequently excluded.

Two reviewers (MRA, PJG) evaluated the methodological quality of individual studies using the Quality Assessment of Diagnostic Accuracy Studies (QUADAS-2) tool [17] and The Joanna Briggs Institute Critical Appraisal tools [18]. Discrepancies were resolved through discussion.

### Statistical analysis

For the primary analysis, we *a priori* planned to use the individual patient as unit of analysis. For the secondary analysis, we used individual eyes as the unit of analysis. We planned to use Review Manager Web version (RevMan Web) to plot estimates of sensitivity and specificity from the studies in receiver operating characteristics (ROC) space and to build forest plots [19]. As the results of the ultrasound scan were expected to be binary, (i.e., presence or absence of orbital cellulitis), we intended to perform meta-analysis using a bivariate model to estimate summary sensitivity (i.e., proportion of individuals with orbital cellulitis who are correctly detected by ultrasound) and specificity (i.e., proportion of individuals without orbital cellulites who are correctly identified by ultrasound) [20]. However, due to insufficient data, we were unable to conduct a meta-analysis. We planned to conduct a meta-analysis if there were sufficient number of studies with low clinical heterogeneity. However, due to limited number of included studies, the results section is primarily descriptive.

Planned sensitivity analyses included the effect of excluding studies where older generations of ultrasound probes were used, and the effect of excluding studies with a high risk of bias for primary outcome. Planned subgroup analyses included the following characteristics: 1) Study type: experimental vs observational; 2) Age: <18 yrs (pediatric) vs. >18 yrs (adult); and 3) Type of ultrasound: standard ultrasound vs. POCUS.

## Results

**Fig 1:**
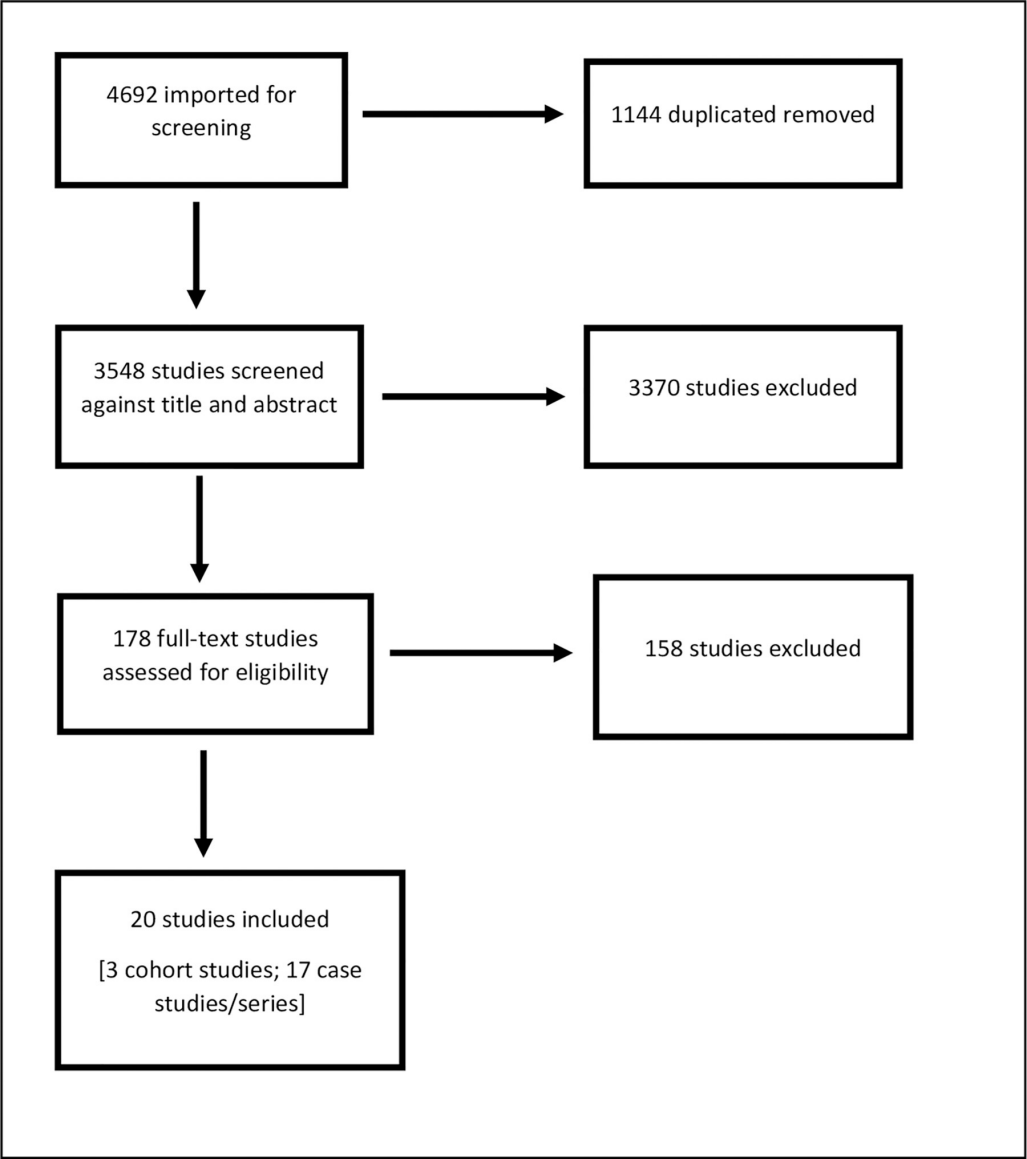
PRISMA flow diagram.

The search yielded 3548 studies, of which 215 underwent full-text screening, and 20 were included. Among these 20 articles, 3 were cohort studies and 17 were case reports/series. The PRISMA diagram in Fig 1 illustrates the process for study selection. Characteristics of the included cohort studies are outlined in S4 Appendix.

### Cohort studies

All three identified retrospective cohort studies were published before 2002, and none reported the sensitivity and specificity of ultrasound. Among the 46 patients reported in these studies, both ultrasound and CT scans were conducted on 18 (39%) cases. A summary of the cohort studies is provided in Table 1. Goodwin et al. was published in 1982 and reviewed medical records of 22 patients diagnosed with orbital cellulitis. Age of the patients ranged from 5 to 68 years. Orbital ultrasound was conducted by experienced technicians and CT scans were reviewed by a neuroradiologist. Findings were in agreement in five of the seven cases where both ultrasound and CT scans were conducted. In the remainder, ultrasound identified wooden foreign body and early abscess formation that were missed in the CT scans. Kaplan et. al. conducted a similar study published in 1999. Seven children between 2 and 13 years old hospitalized with sinus induced orbital infection were examined regularly by an otolaryngologist and ophthalmologist. All children underwent contrast enhanced CT scan and orbital ultrasound by an ophthalmologist within 24 and 48 hours of admission, respectively. Findings from the ultrasound and CT scan were in agreement in three. In two patients with orbital cellulitis (Chandler II) and two patients with Subperiosteal abscess (Chandler III), the authors reported that CT scan could not differentiate between orbital cellulitis and subperiosteal abscess, whereas ultrasound was able to do so. Mair et. al., published in 2002, reviewed clinical records of 17 children aged 1 to 10 years presenting with swelling and erythema of the eyelids. Orbital ultrasound and additional imaging (CT or MRI scan) were performed in only four patients. Although comparison of imaging findings for these patients were not reported, the authors reported that ultrasound identified patients with orbital infection. The authors also suggested that they were able to differentiate between subperiosteal abscess (Chandler III) and subperiosteal inflammatory infiltration (Chandler II) based on the sonographic findings.

**Table 1:** Summary of cohort studies.

Author	Year	Country	Sample (n)	Age (yrs) [mean(range)]	Male: Female	Type of ultrasound	Imaging done^a^	Ultrasound accuracy
Goodwin	1982	USA	22	23 (8–68)	15:7	Routine	7	100%
Kaplan	1999	Israel	7	7.5 (2–13)	4:3	Routine	7	100%
Mair	2002	Austria	17	4.5 (1–10)	10:7	Routine	4	100%^b^

^a^Reported both US and CT/MRI scans

^b^Estimated, not explicitly reported in the source material

**Fig 2:**
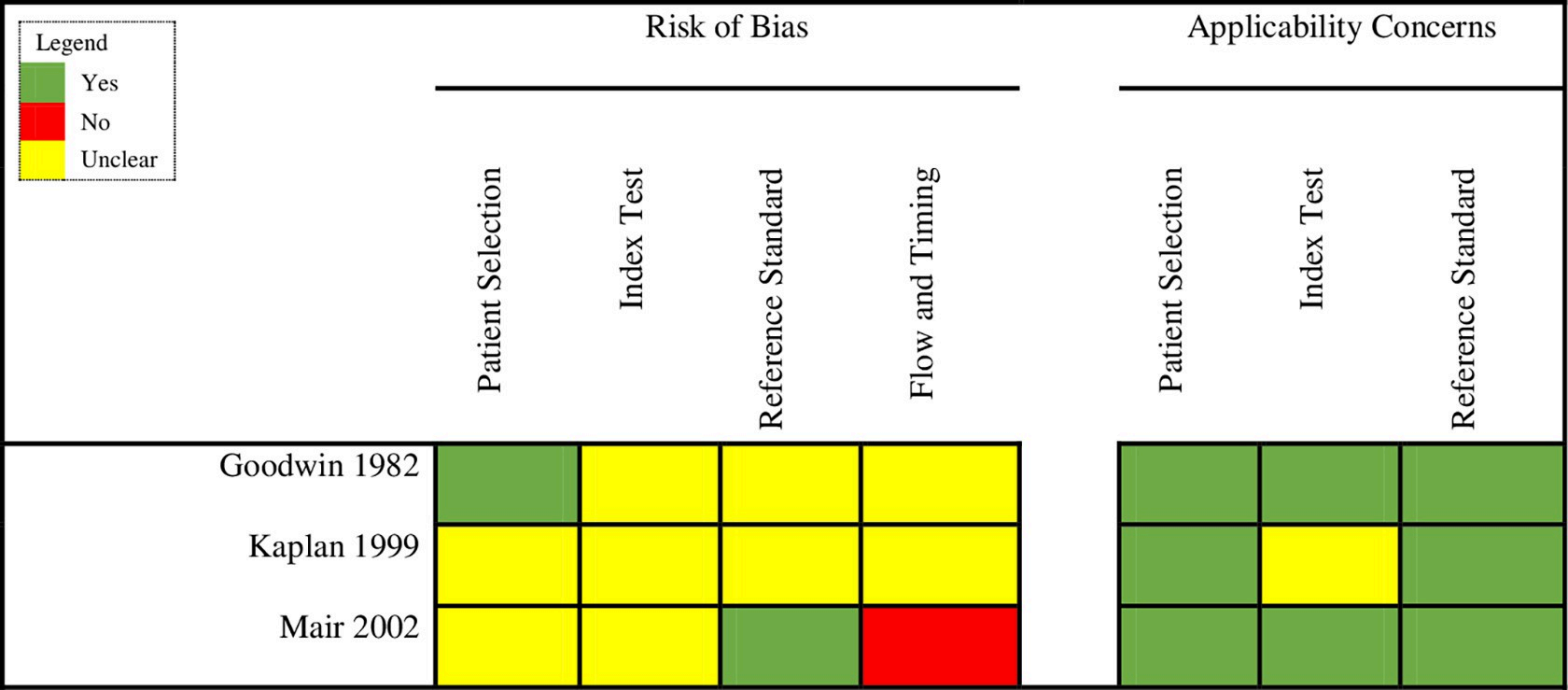
Quality assessment of cohort studies.

QUADAS-2 assessment of the three cohort studies is summarized in Fig 2. For the patient selection domain, there was unclear risk of bias in two studies given the lack of detail on the process of patient selection. For the index test domain, all three studies had an unclear risk of bias as it was unclear from the study methods whether ultrasound (index test) was conducted before or after CT or MRI (reference standard). Risk of bias was low for the reference standard domain in two studies. The studies did not clearly state if the reference interpreters were aware of the index test. Risk of bias for ’flow and timing’ was judged to be high in one study and unclear in the other two. We did not notice any major applicability concerns in any of the studies.

### Case reports/series

There were 17 case reports/series including a total of 23 patients published between 1982 and 2021. Most reports were from hospitals in the USA (n = 8) and included patients ranging from birth to 83 years. A total of 11 males and 11 females were reported in the studies; the sex was not reported in one study. Although a direct comparison between ultrasound and other imaging modalities was not the objective of the studies, the authors report that most ultrasound findings were in agreement with cross-sectional imaging. The Chandler classification was not always specified; however, the authors did acknowledge that the ultrasound findings were sufficient to reach an immediate diagnosis. We have summarized the descriptive findings of case reports in Table 2.

**Table 2:** Summary of case reports.

Author	Year	Country	Sex (Age in yrs)	Type of Ultrasound	Ultrasound findings	CT/MRI (Contrast)	CT/MRI findings
Argelich	2009	Spain	Female (83)	Routine	Retinal detachment with diffuse choroidal thickening.	CT (-)	proptosis, extraocular muscle enlargement, soft tissue swelling, and retrobulbar fat stranding
Beam	2021	USA	Female (21)	Point of care	prominence and edema of the nasal postseptal tissue with echogenic, inflammatory fat.	CT (not reported)	prominence and edema of the nasal postseptal tissue with echogenic, inflammatory fat.
Brooks	2001	USA	Female (12)	Routine	Retrobulbar lesion composed of multiple channels.	CT (not reported)	Soft tissue swelling and inflammatory changes in the right eyelids and orbit. Localized soft tissue inflammatory process in the superomedial orbit and retrobulbar tissues.
Choudhury	2007	India	Female (7)	Routine	indentation of the globe by an echoluscent mass lesion in the intraconal space with extraconal extensions in the inferonasal and superotemporal quadrants.	CT (-)	showed a well-defined homogeneous mass lesion in the intraconal space with extraconal extensions with globe indentation, gas inside the lesion and obliteration of the optic nerve with opacification of the right maxillary sinus
Derr	2012	USA	Female (57)	Routine	well-defined anechoic fluid collection with echogenic borders was seen adjacent to and indenting the inferior most portion of the medial surface of the right orbit	CT (+)	showed extra-orbital inflammation and edema just medial
Harr *Case 1*	1982	USA	Male (70)	Routine	Calcified sclera. Organized, detached retina. Could not image behind globe	CT (not reported)	Pre- and post-septal cellulitis. Phthisis bulbi, no abscess
*Case 2*	1982	USA	Female (9)	Routine	Well-defined low reflective mass superior to globe- abscess & foreign body	CT (not reported)	Extraconal suprabulbar abscess (homogenous enhancement); non-metallic foreign body. Cystic osteomyelitis of orbital roof
*Case 3*	1982	USA	Female (5)	Routine	Retrobulbar mass of mixed echogenicity	CT (not reported)	Intraconal retrobulbar mass (ring enhancement)
*Case 4*	1982	USA	Male (14)	Routine	Well-defined low-reflective mass above globe-abscess Enlarged tenon space Left ethmoid and maxillary sinusitis	CT (not reported)	Extraconal suprabulbar (heterogeneous enhancement) Uveoscleral thickening and enhancement. Left ethmoid and maxillary sinusitis.
*Case 5*	1982	USA	Female (53)	Routine	Well-defined low-reflective mass above globe-abscess Enlarged Tenon space Right frontal sinusitis	CT (not reported)	Abscess superior to globe. Right frontal sinusitis with osteoma blocking drainage.
*Case 6*	1982	USA	Female (18)	Routine	Well-defined low-reflective mass above globe-abscess	CT (not reported)	Abscess superior to globe. Nonmetallic foreign body
Harris	1983	USA	Neonate (sex and age not reported)	Routine	Presence of superior SPA	CT (not reported)	Diffuse inflammation in the left orbit that could not be well localized on axial sections alone. Coronal sections were not accessible. Reconstructed sagittal and coronal projections suggested that the process was limited to the superior orbit.
James	2018	Singapore	Male (13)	US	showed heterogeneous hypoechoic and hyperechoic areas surrounding the orbital septum. posteriorly	CT (+)	showed left periorbital soft tissue thickening and enhancement associated with stranding of intra- and extraconal fat.
Kang	2014	USA	Male (36)	Routine	Showed edema along the anterior aspect of the orbit with nonspecific thickening of the orbital wall	CT (+)	Confirmed the diagnosis of orbital cellulitis
Leccisotti *Case 1*	1996	Not reported	Male (16)	Routine	a well-defined rounded lesion, with low to medium internal reflectivity; on B-scan, the abscess structure was evident	CT (not reported)	a diffuse opacification of paranasal sinuses (bilateral maxillary, sphenoidal and ethmoidal and right cavernous sinus), with orbital and meningeal involvement
*Case 2*	1996	Not reported	Male (41)	Routine	B-scan ultrasound showed a well-defined oval lesion, with very low internal reflectivity	MRI (+)	MRI revealed the mass originated from the left frontal sinus and invaded the orbit through bone erosion.
Manuchehri	2003	UK	Male (6)	Routine	showed increased orbital fat density and volume around the superotemporal orbital rim. There was also a suggestion of hypertrophy of the superior and lateral recta. There was no evidence of thickened sclera and sub-tenon’s space was normal.	CT (-)	The muscle bellies as well as the tendons of the medial and inferior recta were diffusely thickened. This suggested orbital myositis rather than thyroid eye disease.
Okamoto	2009	Japan	Female (60)	Routine	revealed a space-occupying mass lesion in the retrobulbar area that appeared to strongly compress the posterior pole of the eyeball. The right eye was normal in all aspects.	CT (-)	demonstrated an extension of inflammatory mass in the left orbit, frontal and ethmoidal sinusitis accompanying destruction of the orbital bones, capsular formation of the tumor, and inflammation in the subperiosteal region.
Osuagwu	2011	Nigeria	Male (15)	Routine	Demonstrated marked thickening of the right upper eyelid and an oval hypoechoic lesion with internal low level echoes suggestive of an abscess was seen in the retro-orbital space medially. Both eyeballs appeared normal	CT (not reported)	multiple, fairly rounded, ring- enhancing hypodense masses in the right orbit and sub-palpebral tissue with resulting in proptosis of the right globe. The superior oblique and lateral rectus muscles were enlarged and there was thickening of the pre-orbital tissue.
Schellini	2019	Saudi Arabia	Male (18)	Routine	Not reported Ultrasound B-scan OS was unremarkable.	CT (+)	Showed left periorbital and pre-septal soft tissue thickening and stranding of the pre-septal fat planes associated with enlargement of the left lacrimal gland [pre-septal and orbital cellulitis)
Seguin	2019	Canada	Male (5)	Point-of-care	a hypoechoic collection with a hyperechoic rim was identified in the medial retro-orbital fat of the right orbit. A diagnosis of orbital abscess was suspected	CT (not reported	a 3 x 1-cm right medial orbital abscess
Sty	1980	USA	Male (10)	Routine	Abnormal fluid collection medial to the globe	CT (not reported)	Abnormal fluid collection medial to the globe
Allen	1985	USA	Female (57)	US	no evidence of postseptal orbital inflammation, and sinus films were normal. Preseptal cellulitis was diagnosed, enlargement of all rectus muscles and prolongation of the posterior orbital wave pattern. An area of focal echolucency within the orbital fat pattern was detected immediately posterior to the lacrimal sac	CT (+)	Localized enhancement in addition to fluid in the adjacent ethmoidal sinus.

The critical appraisal of case reports revealed that most of the cases were well reported. The authors defined the patient demographics and described the diagnostic tests clearly. Only three of thirteen studies did not clearly describe the outcome of the patient post intervention. Critical appraisal of the four case-series identified major concerns. It was either unclear or the authors did not report if consecutive patients were included and case inclusion were unclear. However, patient demographics, measurement of the clinical condition, and follow up were clearly reported. The appraisal of the case reports and case-series are presented in Tables 3 and 4.

**Table 3:** Joanna Briggs institute critical appraisal checklist for case reports.

Q #	Critical appraisal checklist	Yes	No	Unclear	N/A
Q1	Were patient’s demographic characteristics clearly described?	√(13)			
Q2	Was the patient’s history clearly described and presented as a timeline?	√(13)			
Q3	Was the current clinical condition of the patient on presentation clearly described?	√(13)			
Q4	Were diagnostic tests or assessment methods and the results clearly described?	√(13)			
Q5	Was the intervention(s) or treatment procedure(s) clearly described?	√(11)	√(2)		
Q6	Was the post-intervention clinical condition clearly described?	√(10)	√(3)		
Q7	Were adverse events (harms) or unanticipated events identified and described?	√(10)	√(3)		
Q8	Does the case report provide takeaway lessons?	√(13)			

**Table 4:** Joanna Briggs institute critical appraisal checklist for case series.

Q #	Critical appraisal checklist	Yes	No	Unclear	N/A
Q1	Were there clear criteria for inclusion in the case series?			√(4)	
Q2	Was the condition measured in a standard, reliable way for all participants included in the case series?	√(4)			
Q3	Were valid methods used for identification of the condition for all participants included in the case series?	√(4)			
Q4	Did the case series have consecutive inclusion of participants?		√(3)	√(1)	
Q5	Did the case series have complete inclusion of participants?		√(3)	√(1)	
Q6	Was there clear reporting of the demographics of the participants in the study?	√(4)			
Q7	Was there clear reporting of clinical information of the participants?	√(3)	√(1)		
Q8	Were the outcomes or follow up results of cases clearly reported?	√(4)			
Q9	Was there clear reporting of the presenting site(s)/clinic(s) demographic information?	√(3)		√(1)	
Q10	Was statistical analysis appropriate?				√(4)

### Guidance on ultrasound technique

Some of the studies reported the brand, model, and frequency of the transducer used to examine the patients, but it was difficult to compare or reach conclusions due to substantial variation across the ultrasound devices. Earlier studies do however suggest that a high-frequency (>10 Mhz) linear array transducer could be used to examine the orbital region [9, 10]. While the studies provided limited information on diagnostic performance, the authors report that most of the patients were examined in supine position without sedation. Some techniques were suggested that could guide better orbital assessment with ultrasound. For instance, the authors suggested to place the transducer on the closed upper eyelid using a non-irritating gel and to avoid any pressure on the eyeball and orbital tissues [10, 21, 22]. A transparent adhesive dressing (Tegaderm) could also be gently applied over the closed eye before applying the gel, ensuring no air is trapped between the plastic and the skin [22].

The medial orbital wall can be visualized by moving the transducer as far as possible toward the lateral canthus of the eye [10]. Another author pointed out that, while imaging the eye in the standard plane using POCUS, no obvious anomaly was detected. However, by rocking the probe medially and laterally and focusing on the nasal and temporal tissues additional views could be obtained. This alternating motion revealed prominence and edema of the nasal post septal tissue with echogenic, inflammatory fat in an adult female patient with orbital cellulitis [23]. For the transverse and longitudinal planes, the marker of the transducer can be oriented towards the right or head of the patient, respectively. The transducer can also be fanned back and forth for clear visibility of anterior and posterior anatomy of the eye. For assessing extraocular muscles, the patient can be asked to move their eye or fix their gaze on objects. The same study suggested that ocular POCUS could be performed while distracting the child by a video on the parent’s smartphone [22]. Studies have also suggested against use of color flow or doppler mode to limit the theoretical risk of damaging tissues [24, 25]. A table listing all the ultrasound techniques have been provided in the S5 Appendix.

## Discussion

This systematic review of the diagnostic test accuracy of orbital ultrasound to diagnose orbital cellulitis found limited, low-quality evidence of comparable performance to conventional cross-sectional imaging. Only 3 cohort and 17 case reports/series were identified; there were no prospective observational studies or randomized controlled trials. Furthermore, included studies were several decades old and used orbital ultrasound mainly as a supplementary imaging modality. Despite the limitations, the studies highlight the potential value of orbital ultrasound in both diagnosis and management of severe orbital infections [8, 10].

Several authors noted the benefit in choosing ultrasound as it is portable and accessible and does not require sedation in pediatric patients [10]. The diagnostic workup is rapid which can shorten the interval between clinical presentation and initiation of treatment, especially in patients with non-specific clinical findings. Orbital cellulitis is considered an ophthalmologic emergency which requires urgent antibiotic therapy, which if left untreated, or if treatment is delayed, can be both sight- and life-threatening. In this clinical scenario, a cost-effective, rapid, low risk, bedside procedure such as ultrasound might be useful. Ultrasound may be especially useful in young children and uncooperative patients who would require sedation or a general anesthetic [22]. Another benefit of ultrasound is its use for reassessment and evaluation of response to treatment [8, 21, 26]. Compared with CT scan, ultrasound provides useful information but without radiation exposure and with less cost [8]. Furthermore, if ultrasound can differentiate pre and post septal infections, this may eliminate the need for additional cross-sectional imaging with CT or MRI.

POCUS can be performed at the bedside, does not require sedation, and is especially useful in children [27]. Pediatric emergency medicine has recognized the potential of POCUS to improve care for their patients [28]. POCUS is also gaining popularity in the inpatient setting. Its benefits in clinical decision-making and patient management are increasingly recognized [29]. For example, medical schools have also started incorporating POCUS into their undergraduate medical education [30]. POCUS is now being used to detect cranial hemorrhage in preterm infants [27] and skull fractures in toddlers [31]. POCUS has also been found to be beneficial in investigating orbital trauma as it can identify orbital fractures and correlates well with CT [32]. POCUS demonstrates a high sensitivity and specificity in the diagnosis of traumatic eye injury, reporting an 85% sensitivity, 98% specificity, and 97% accuracy in detection of traumatic lens dislocation [33]. Optic nerve sheath diameter may be measured accurately with transorbital ultrasound for the detection of raised intra-cranial pressure or diagnosis of papilledema [34–37]. POCUS is also used to facilitate interventions safely, such as obtaining vascular access and endotracheal intubation in children [27]. Ultrasound is not limited to making initial diagnoses, but is also used to evaluate response to treatments [38, 39].

Like any diagnostic test, orbital ultrasound has limitations. While it may differentiate between pre- and post-septal infection, it cannot sufficiently assess the orbital apex and the paranasal sinuses. It also cannot define the abscesses of the posterior orbit, nor can it assess the intracranial extension of orbital infection, which is an important case of morbidity [9]. Patients who fail to improve with appropriate care, those with neurologic symptoms, or showing signs of clinical deterioration, should still undergo a CT or MRI scan as part of complete diagnostic workup.

### Limitations

Our systematic review has several limitations. First, we had to exclude 37 studies that were awaiting classification as we could not obtain the full-text despite multiple attempts at contacting study authors and reference libraries. Most of these studies were completed several decades ago. Second, the exact timing of the reference standard test was often unclear given that most studies were not designed to compare ultrasound to other imaging modalities. Third, several included studies were decades old, and the imaging technologies might not be representative of those currently used. Fourth, many of the included studies were of low quality with a high risk of bias; no prospective, blinded, diagnostic accuracy studies were identified. However, such studies are feasible given similar studies in orbital ultrasound for retinal detachment in adult care [12, 40]. Studies on the application of ultrasound are susceptible to publication bias, as studies that report utility of a diagnostic tool are more likely to be published compared with studies that report it is not useful.

While the evidence generated from this review is limited, it is the first to systematically assess the benefit of ultrasound compared other imaging modalities to diagnose orbital cellulitis. Our findings support the need for future prospective observational studies or trials to evaluate the diagnostic test accuracy of ultrasound to diagnose and evaluate response to treatments in orbital cellulitis.

## Supporting information

S1 Appendix PRISMA-DTA checklist. (DOC)

S2 Appendix Search strategy. (DOCX)

S3 Appendix Data extraction template. (DOCX)

S4 Appendix Characteristics of included cohort studies. (DOCX)

S5 Appendix Table listing various ultrasound techniques. (DOCX)

## References

[1] Gill PJ, Drouin O, Pound C, Quet J, Wahi G, Bayliss A, et al. Factors associated with surgery and imaging characteristics in severe orbital infections. J Pediatr. 2022 Sep;248:66–73.e7. doi: 10.1016/j.jpeds.2022.05.010 35568061

[2] Eustis HS, Mafee MF, Walton C, Mondonca J. MR imaging and CT of orbital infections and complications in acute rhinosinusitis. Radiologic Clinics of North America. 1998 Nov 1;36(6):1165–83. doi: 10.1016/s0033-8389(05)70238-4 9884695

[3] Hauser JA, Taylor AM, Pandya B. How to image the adult patient with Fontan circulation. Circulation: Cardiovascular Imaging. 2017 May;10(5):e004273. doi: 10.1161/CIRCIMAGING.116.004273 28495823

[4] Krueger C, Mahant S, Begum N, Widjaja E, Science M, Parkin PC, et al. Changes in the management of severe orbital infections over seventeen years. Hospital Pediatrics. 2021 Jun;11(6):613–21. doi: 10.1542/hpeds.2020-001818 34031136

[5] Jyani R, Ranade D, Joshi P. Spectrum of orbital cellulitis on magnetic resonance imaging. Cureus. 2020 Aug 11;12(8). doi: 10.7759/cureus.9663 32923259 PMC7485920

[6] Cornelius RS. Magnetic resonance imaging of the head and Neck. Topics in Magnetic Resonance Imaging. 1999 Dec 1;10(6):347. 10.1097/00002142-199912000-00002 10643879

[7] Blaivas M, Theodoro D, Sierzenski PR. A study of bedside ocular ultrasonography in the emergency department. Academic emergency medicine. 2002 Aug;9(8):791–9. doi: 10.1111/j.1553-2712.2002.tb02166.x 12153883

[8] Goodwin WJ Jr, Weinshall M, Chandler JR. The role of high resolution computerized tomography and standardized ultrasound in the evaluation of orbital cellulitis. The Laryngoscope. 1982 Jul;92(7):728–31. 7087639

[9] Kaplan DM, Briscoe D, Gatot A, Niv A, Leiberman A, Fliss DM. The use of standardized orbital ultrasound in the diagnosis of sinus induced infections of the orbit in children: a preliminary report. International Journal of Pediatric Otorhinolaryngology. 1999 May 5;48(2):155–62. doi: 10.1016/s0165-5876(99)00023-3 10375041

[10] Mair MH, Geley T, Judmaier W, Gaßner I. Using orbital sonography to diagnose and monitor treatment of acute swelling of the eyelids in pediatric patients. American Journal of Roentgenology. 2002 Dec;179(6):1529–34. doi: 10.2214/ajr.179.6.1791529 12438049

[11] Maw AM, Huebschmann AG, Mould-Millman NK, Dempsey AF, Soni NJ. Point-of-care ultrasound and modernization of the bedside assessment. Journal of graduate medical education. 2020 Dec;12(6):661–5. doi: 10.4300/JGME-D-20-00216.1 33391586 PMC7771602

[12] Gottlieb M, Holladay D, Peksa GD. Point‐of‐care ocular ultrasound for the diagnosis of retinal detachment: a systematic review and meta‐analysis. Academic Emergency Medicine. 2019 Aug;26(8):931–9. doi: 10.1111/acem.13682 30636351

[13] Lahham, S, Shniter, I., Thompson, M., Le, D., Chadha, T., Mailhot, T., et al. Point-of-care ultrasonography in the diagnosis of retinal detachment, vitreous hemorrhage, and vitreous detachment in the emergency department. JAMA network open, 2(4), pp.e192162-e192162. 10.1001/jamanetworkopen.2019.2162 PMC6481597 30977855

[14] McInnes MD, Moher D, Thombs BD, McGrath TA, Bossuyt PM, Clifford T, et al. Preferred reporting items for a systematic review and meta-analysis of diagnostic test accuracy studies: the PRISMA-DTA statement. Jama. 2018 Jan 23;319(4):388–96. doi: 10.1001/jama.2017.19163 29362800

[15] Veritas Health Innovation. Covidence systematic review software.

[16] Chandler JR, Langenbrunner DJ, Stevens ER. The pathogenesis of orbital complications in acute sinusitis. The Laryngoscope. 1970 Sep;80(9):1414–28. doi: 10.1288/00005537-197009000-00007 5470225

[17] Whiting PF, Rutjes AW, Westwood ME, Mallett S, Deeks JJ, Reitsma JB, et al. QUADAS-2: a revised tool for the quality assessment of diagnostic accuracy studies. Annals of internal medicine. 2011 Oct 18;155(8):529–36. doi: 10.7326/0003-4819-155-8-201110180-00009 22007046

[18] Moola S, Munn Z, Tufanaru C, Aromataris E, Sears K, Sfetcu R, et al. Chapter 7: Systematic reviews of etiology and risk. Joanna briggs institute reviewer’s manual. The Joanna Briggs Institute. 2017 Jul 17;5.

[19] Cochrane Collaboration. Review Manager Web (RevMan Web).

[20] Reitsma JB, Glas AS, Rutjes AW, Scholten RJ, Bossuyt PM, Zwinderman AH. Bivariate analysis of sensitivity and specificity produces informative summary measures in diagnostic reviews. Journal of clinical epidemiology. 2005 Oct 1;58(10):982–90. doi: 10.1016/j.jclinepi.2005.02.022 16168343

[21] Derr C, Shah A. Bedside ultrasound in the diagnosis of orbital cellulitis and orbital abscess. Emergency radiology. 2012 Jun;19(3):265–7. doi: 10.1007/s10140-011-0993-0 22322465

[22] Seguin J, Le CK, Fischer JW, Tessaro MO, Berant R. Ocular point-of-care ultrasound in the pediatric emergency department. Pediatric emergency care. 2019 Mar 1;35(3):e53–8. doi: 10.1097/PEC.0000000000001762 30822281

[23] Beam G, Check R, Denne N, Minardi J, End B. Point-of-Care Ultrasound Findings in a Case of Orbital Cellulitis: A Case Report. The Journal of Emergency Medicine. 2021 Aug 1;61(2):157–60. doi: 10.1016/j.jemermed.2021.03.033 33972132

[24] Miller DL, Smith NB, Bailey MR, Czarnota GJ, Hynynen K, Makin IR, Bioeffects Committee of the American Institute of Ultrasound in Medicine. Overview of therapeutic ultrasound applications and safety considerations. Journal of ultrasound in medicine. 2012 Apr;31(4):623–34. 22441920 10.7863/jum.2012.31.4.623 PMC3810427

[25] Lawrence JP. Physics and instrumentation of ultrasound. Critical care medicine. 2007 Aug 1;35(8):S314–22. doi: 10.1097/01.CCM.0000270241.33075.60 17667455

[26] James V, Ong GY. Elevated optic disc height and increased optic nerve sheath diameter on bedside ultrasound in a pediatric patient with orbital cellulitis: more than meets the eye. The Journal of Emergency Medicine. 2018 Dec 1;55(6):813–6. doi: 10.1016/j.jemermed.2018.08.006 30253955

[27] Ichihashi K, Nonaka K. Point-of-care ultrasound for children. Journal of Medical Ultrasonics. 2022 Jan 21:1–6. doi: 10.1007/s10396-021-01169-0 35059920

[28] Vieira RL, Hsu D, Nagler J, Chen L, Gallagher R, Levy JA. Pediatric emergency medicine fellow training in ultrasound: consensus educational guidelines. Academic Emergency Medicine. 2013 Mar;20(3):300–6. doi: 10.1111/acem.12087 23517263

[29] Cid-Serra X, Hoang W, El-Ansary D, Canty D, Royse A, Royse C. Clinical Impact of Point-of-Care Ultrasound in Internal Medicine Inpatients: A Systematic Review. Ultrasound in Medicine & Biology. 2021 Nov 2. doi: 10.1016/j.ultrasmedbio.2021.09.013 34740496

[30] Steinmetz P, Dobrescu O, Oleskevich S, Lewis J. Bedside ultrasound education in Canadian medical schools: a national survey. Canadian Medical Education Journal. 2016;7(1):e78. 27103956 PMC4830376

[31] Dehbozorgi A, Mousavi-Roknabadi RS, Hosseini-Marvast SR, Sharifi M, Sadegh R, Farahmand F, et al. Diagnosing skull fracture in children with closed head injury using point-of-care ultrasound vs. computed tomography scan. European Journal of Pediatrics. 2021 Feb;180(2):477–84. doi: 10.1007/s00431-020-03851-w 33118087 PMC7594935

[32] Ojaghihaghighi S, Lombardi KM, Davis S, Vahdati SS, Sorkhabi R, Pourmand A. Diagnosis of traumatic eye injuries with point-of-care ocular ultrasonography in the emergency department. Annals of emergency medicine. 2019 Sep 1;74(3):365–71. doi: 10.1016/j.annemergmed.2019.02.001 30905470

[33] Haghighi SH, Begi HR, Sorkhabi R, Tarzamani MK, Zonouz GK, Mikaeilpour A, et al. Diagnostic accuracy of ultrasound in detection of traumatic lens dislocation. Emergency. 2014;2(3):121. 26495362 PMC4614573

[34] Mohson KI, Auday N. Role of orbital ultrasound in the assessment of clinically detected papilledema. Journal of Medical Ultrasound. 2019 Jul;27(3):135. doi: 10.4103/JMU.JMU_70_18 31867176 PMC6905264

[35] Ertl M, Schlachetzki F, Gamulescu MA. Application of orbital sonography in neurology. INTECH Open Access Publisher; 2012 Feb 3.

[36] Carter SB, Pistilli M, Livingston KG, Gold DR, Volpe NJ, Shindler KS, et al. The role of orbital ultrasonography in distinguishing papilledema from pseudopapilledema. Eye. 2014 Dec;28(12):1425–30. doi: 10.1038/eye.2014.210 25190532 PMC4268455

[37] Qayyum H, Ramlakhan S. Can ocular ultrasound predict intracranial hypertension? A pilot diagnostic accuracy evaluation in a UK emergency department. European Journal of Emergency Medicine. 2013 Apr 1;20(2):91–7. 22327166 10.1097/MEJ.0b013e32835105c8

[38] Maaser C, Petersen F, Helwig U, Fischer I, Roessler A, Rath S, et al. Intestinal ultrasound for monitoring therapeutic response in patients with ulcerative colitis: results from the TRUST&UC study. Gut. 2020 Sep 1;69(9):1629–36. 31862811 10.1136/gutjnl-2019-319451 PMC7456734

[39] Bélard S, Heuvelings CC, Banderker E, Bateman L, Heller T, Andronikou S, et al. Utility of point-of-care ultrasound in children with pulmonary tuberculosis. The Pediatric infectious disease journal. 2018 Jul;37(7):637. doi: 10.1097/INF.0000000000001872 29278611 PMC5995614

[40] Vrablik ME, Snead GR, Minnigan HJ, Kirschner JM, Emmett TW, Seupaul RA. The diagnostic accuracy of bedside ocular ultrasonography for the diagnosis of retinal detachment: a systematic review and meta-analysis. Annals of emergency medicine. 2015 Feb 1;65(2):199–203. doi: 10.1016/j.annemergmed.2014.02.020 24680547

