# Supplementary material for: Diagnostic test accuracy of ultrasound for orbital cellulitis: A systematic review
Source: PLoS One. 2023 Jul 6;18(7):e0288011. doi: 10.1371/journal.pone.0288011 (PMC10325084; doi:10.1371/journal.pone.0288011)
Supplement: S2 Appendix — (DOCX) [file pone.0288011.s002.docx]

**S2 Appendix B: Search strategy**

| **Database [Platform]** *Searches run* | **Results** |
| --- | --- |
| MEDLINE(R) and Epub Ahead of Print, In-Process & Other Non-Indexed Citations and Daily [OVID] 1946 to July 02, 2021 | 1198 |
| Embase Classic+Embase [OVID] 1947 to 2021 Week 26 | 2621 |
| Cochrane Central Register of Controlled Trials [OVID] May 2021 | 65 |
| Web of Science [Clarivate Analytics] | 404 |
| **TOTAL** | **4,288** |

**Ovid MEDLINE(R) and Epub Ahead of Print, In-Process, In-Data-Review & Other Non-Indexed Citations and Daily**1946 to July 02, 2021
Search Strategy:

| **#** | **Searches** | **Results** |
| --- | --- | --- |
| 1 | exp Cellulitis/ | 8529 |
| 2 | Orbital Cellulitis/ | 537 |
| 3 | ((periorbit* or orbit* or preseptal or "pre septal" or postseptal or "post septal") adj3 celluliti*).tw,kf. | 1683 |
| 4 | ((subperiosteal or "sub periosteal" or orbital or intraconal) adj1 abscess*).tw,kf. | 848 |
| 5 | exp Eye Infections, Bacterial/ | 13745 |
| 6 | (Bacterial adj3 eye infect*).tw,kf. | 36 |
| 7 | Orbital Diseases/ | 5835 |
| 8 | (orbital adj1 disease*).tw,kf. | 813 |
| 9 | exp Sinusitis/ | 21482 |
| 10 | (sinusitis or (sinus adj2 infect*) or rhinosinusitis).tw,kf. | 26126 |
| 11 | "Sinus venous thrombosis".tw,kf. | 181 |
| 12 | (orbital adj2 inflamm*).tw,kf. | 1180 |
| 13 | or/1-12 | 60018 |
| 14 | exp Ultrasonography/ | 455180 |
| 15 | (ultrasound? or ultrasonograph* or echograph* or echotomograph* or medical sonography or ultrasonic diagnos#s or ultrasonic imag* or ultrasonic tomograph* or ultrasonographic imag* or POCUS or "POC US" or "POC USG").tw,kf. | 369101 |
| 16 | or/14-15 | 631413 |
| 17 | 13 and 16 | 1198 |

**Embase Classic+Embase**1947 to 2021 Week 26
Search Strategy:

| **#** | **Searches** | **Results** |
| --- | --- | --- |
| 1 | cellulitis/ | 22973 |
| 2 | orbit cellulitis/ | 2343 |
| 3 | ((periorbit* or orbit* or preseptal or 'pre septal' or postseptal or 'post septal') adj3 celluliti*).tw,kw. | 2226 |
| 4 | ((subperiosteal or 'sub periosteal' or orbital or intraconal) adj1 abscess*).tw,kw. | 1207 |
| 5 | exp bacterial eye infection/ | 9289 |
| 6 | (Bacterial adj3 eye infect*).tw,kw. | 82 |
| 7 | orbit disease/ | 4526 |
| 8 | (orbital adj1 disease*).tw,kw. | 1132 |
| 9 | (sinusitis or (sinus adj2 infect*) or rhinosinusitis).tw,kw. | 37150 |
| 10 | exp sinusitis/ | 50276 |
| 11 | (sinusitis or (sinus adj2 infect*) or rhinosinusitis).tw,kw. | 37150 |
| 12 | Sinus venous thrombosis.tw,kw. | 395 |
| 13 | (orbital adj2 inflamm*).tw,kw. | 1540 |
| 14 | or/1-13 | 96045 |
| 15 | exp echography/ | 855257 |
| 16 | (ultrasound? or ultrasonograph* or echograph* or echotomograph* or medical sonography or ultrasonic diagnos#s or ultrasonic imag* or ultrasonic tomograph* or ultrasonographic imag* or POCUS or "POC US" or "POC USG").tw,kw. | 565907 |
| 17 | or/15-16 | 1125633 |
| 18 | 14 and 17 | 3284 |
| 19 | limit 18 to (books or chapter or conference abstract or conference paper or "conference review") | 663 |
| 20 | 18 not 19 | 2621 |

**Cochrane Central Register of Controlled Trials**May 2021
Search Strategy:

| **#** | **Searches** | **Results** |
| --- | --- | --- |
| 1 | Cellulitis/ | 174 |
| 2 | Orbital Cellulitis/ | 2 |
| 3 | ((periorbit* or orbit* or preseptal or "pre septal" or postseptal or "post septal") adj3 celluliti*).tw,kw. | 21 |
| 4 | ((subperiosteal or "sub periosteal" or orbital or intraconal) adj1 abscess*).tw,kw. | 1 |
| 5 | exp Eye Infections, Bacterial/ | 507 |
| 6 | (Bacterial adj3 eye infect*).tw,kw. | 14 |
| 7 | Orbital Diseases/ | 20 |
| 8 | (orbital adj1 disease*).tw,kw. | 14 |
| 9 | exp Sinusitis/ | 1025 |
| 10 | (sinusitis or (sinus adj2 infect*) or rhinosinusitis).tw,kw. | 3427 |
| 11 | Sinus venous thrombosis.tw,kw. | 4 |
| 12 | (orbital adj2 inflamm*).tw,kw. | 14 |
| 13 | or/1-12 | 4326 |
| 14 | exp ultrasonography/ | 14359 |
| 15 | (ultrasound? or ultrasonograph* or echograph* or echotomograph* or medical sonography or ultrasonic diagnos#s or ultrasonic imag* or ultrasonic tomograph* or ultrasonographic imag* or POCUS or "POC US" or "POC USG").tw,kw. | 42549 |
| 16 | or/14-15 | 49856 |
| 17 | 13 and 16 | 65 |

**Web of Science [Clarivate Analytics**

**July 5, 2021**

| **Set** | **Results** | **Searches** |
| --- | --- | --- |
| **10** | **404** | #9 AND #8  *Indexes=SCI-EXPANDED, SSCI, ESCI Timespan=All years* |
| **9** | **449,589** | TS=(ultrasound* or ultrasonograph* or echograph* or echotomograph* or "medical sonography" or "ultrasonic diagnosis" or "ultrasonic diagnoses" or "ultrasonic imag*" or "ultrasonic tomograph*" or "ultrasonographic imag*" or POCUS or "POC US" or "POC USG")  *Indexes=SCI-EXPANDED, SSCI, ESCI Timespan=All years* |
| **8** | **28,500** | #7 OR #6 OR #5 OR #4 OR #3 OR #2 OR #1  *Indexes=SCI-EXPANDED, SSCI, ESCI Timespan=All years* |
| **7** | **1,251** | TS=(orbital NEAR/2 inflamm*)  *Indexes=SCI-EXPANDED, SSCI, ESCI Timespan=All years* |
| **6** | **233** | TS="Sinus venous thrombosis"  *Indexes=SCI-EXPANDED, SSCI, ESCI Timespan=All years* |
| **5** | **24,956** | TS=(sinusitis or rhinosinusitis) OR TS= (sinus NEAR/2 infect*)  *Indexes=SCI-EXPANDED, SSCI, ESCI Timespan=All years* |
| **4** | **1,011** | TS=(orbital NEAR/1 disease*)  *Indexes=SCI-EXPANDED, SSCI, ESCI Timespan=All years* |
| **3** | **47** | TS=(Bacterial NEAR/3 eye infect*)  *Indexes=SCI-EXPANDED, SSCI, ESCI Timespan=All years* |
| **2** | **823** | TS=((subperiosteal or "sub periosteal" or orbital or intraconal) NEAR/1 abscess*)  *Indexes=SCI-EXPANDED, SSCI, ESCI Timespan=All years* |
| **1** | **1,596** | TS=((periorbit* or orbit* or preseptal or "pre septal" or postseptal or "post septal") NEAR/3 celluliti*)  *Indexes=SCI-EXPANDED, SSCI, ESCI Timespan=All years* |

TS=((periorbit* or orbit* or preseptal or "pre septal" or postseptal or "post septal") NEAR/3 celluliti*)

TS=((subperiosteal or "sub periosteal" or orbital or intraconal) NEAR/1 abscess*)

TS=(Bacterial NEAR/3 eye infect*)

TS=(orbital NEAR/1 disease*)

TS=(sinusitis or rhinosinusitis) OR TS= (sinus NEAR/2 infect*)

TS="Sinus venous thrombosis"

TS=(orbital NEAR/2 inflamm*)

Updated search strategy (July 02, 2021 to August 10, 2022)

| **Database [Platform]** *Search update run August 10, 2022* | **Results** |
| --- | --- |
| MEDLINE(R) and Epub Ahead of Print, In-Process & Other Non-Indexed Citations and Daily [OVID] 1946 to August 09, 2022 | 59 |
| Embase Classic+Embase <1947 to 2022 Week 31> | 308 |
| Cochrane Central Register of Controlled Trials [OVID] July 2022 | 2 |
| Web of Science [Clarivate Analytics] August 10 2022 | 35 |
| **TOTAL** | **404** |

**Ovid MEDLINE(R) and Epub Ahead of Print, In-Process, In-Data-Review & Other Non-Indexed Citations and Daily**1946 to August 10, 2022
Search Strategy:

| **#** | **Searches** | **Results** |
| --- | --- | --- |
| 1 | exp Cellulitis/ | 8848 |
| 2 | Orbital Cellulitis/ | 618 |
| 3 | ((periorbit* or orbit* or preseptal or "pre septal" or postseptal or "post septal") adj3 celluliti*).tw,kf. | 1836 |
| 4 | ((subperiosteal or "sub periosteal" or orbital or intraconal) adj1 abscess*).tw,kf. | 905 |
| 5 | exp Eye Infections, Bacterial/ | 14299 |
| 6 | (Bacterial adj3 eye infect*).tw,kf. | 43 |
| 7 | Orbital Diseases/ | 6086 |
| 8 | (orbital adj1 disease*).tw,kf. | 885 |
| 9 | exp Sinusitis/ | 22626 |
| 10 | (sinusitis or (sinus adj2 infect*) or rhinosinusitis).tw,kf. | 27732 |
| 11 | "Sinus venous thrombosis".tw,kf. | 221 |
| 12 | (orbital adj2 inflamm*).tw,kf. | 1277 |
| 13 | or/1-12 | 62922 |
| 14 | exp Ultrasonography/ | 477491 |
| 15 | (ultrasound? or ultrasonograph* or echograph* or echotomograph* or medical sonography or ultrasonic diagnos#s or ultrasonic imag* or ultrasonic tomograph* or ultrasonographic imag* or POCUS or "POC US" or "POC USG").tw,kf. | 396735 |
| 16 | 14 or 15 | 667601 |
| 17 | 13 and 16 | 1247 |
| 18 | limit 17 to ez=20210702-20221231 | 33 |
| 19 | limit 17 to ed=20210702-20221231 | 49 |
| 20 | limit 17 to dt=20210702-20221231 | 32 |
| 21 | 18 or 19 or 20 | 59 |

Embase Classic+Embase <1947 to 2022 Week 31>
Search Strategy:

| **#** | **Searches** | **Results** |
| --- | --- | --- |
| 1 | cellulitis/ | 24397 |
| 2 | orbit cellulitis/ | 2588 |
| 3 | ((periorbit* or orbit* or preseptal or 'pre septal' or postseptal or 'post septal') adj3 celluliti*).tw,kw. | 2334 |
| 4 | ((subperiosteal or 'sub periosteal' or orbital or intraconal) adj1 abscess*).tw,kw. | 1226 |
| 5 | exp bacterial eye infection/ | 9588 |
| 6 | (Bacterial adj3 eye infect*).tw,kw. | 72 |
| 7 | orbit disease/ | 4774 |
| 8 | (orbital adj1 disease*).tw,kw. | 1066 |
| 9 | (sinusitis or (sinus adj2 infect*) or rhinosinusitis).tw,kw. | 38250 |
| 10 | exp sinusitis/ | 53144 |
| 11 | Sinus venous thrombosis.tw,kw. | 441 |
| 12 | (orbital adj2 inflamm*).tw,kw. | 1579 |
| 13 | or/1-12 | 100946 |
| 14 | exp echography/ | 927781 |
| 15 | (ultrasound? or ultrasonograph* or echograph* or echotomograph* or medical sonography or ultrasonic diagnos#s or ultrasonic imag* or ultrasonic tomograph* or ultrasonographic imag* or POCUS or "POC US" or "POC USG").tw,kw. | 597570 |
| 16 | or/14-15 | 1211110 |
| 17 | 13 and 16 | 3625 |
| 18 | limit 17 to (books or chapter or conference abstract or conference paper or "conference review") | 748 |
| 19 | 17 not 18 | 2877 |
| 20 | limit 19 to dc=20210702-20221231 | 308 |
| 21 | limit 19 to dd=20210702-20221231 | 18 |
| 22 | 20 or 21 | 308 |
| 23 | remove duplicates from 22 | 308 |

**Cochrane Central Register of Controlled Trials July 2022**
Search Strategy:

| **#** | **Searches** | **Results** |
| --- | --- | --- |
| 1 | Cellulitis/ | 180 |
| 2 | Orbital Cellulitis/ | 2 |
| 3 | ((periorbit* or orbit* or preseptal or "pre septal" or postseptal or "post septal") adj3 celluliti*).tw,kw. | 21 |
| 4 | ((subperiosteal or "sub periosteal" or orbital or intraconal) adj1 abscess*).tw,kw. | 1 |
| 5 | exp Eye Infections, Bacterial/ | 517 |
| 6 | (Bacterial adj3 eye infect*).tw,kw. | 13 |
| 7 | Orbital Diseases/ | 21 |
| 8 | (orbital adj1 disease*).tw,kw. | 13 |
| 9 | exp Sinusitis/ | 1111 |
| 10 | (sinusitis or (sinus adj2 infect*) or rhinosinusitis).tw,kw. | 3665 |
| 11 | Sinus venous thrombosis.tw,kw. | 4 |
| 12 | (orbital adj2 inflamm*).tw,kw. | 12 |
| 13 | or/1-12 | 4584 |
| 14 | exp ultrasonography/ | 15202 |
| 15 | (ultrasound? or ultrasonograph* or echograph* or echotomograph* or medical sonography or ultrasonic diagnos#s or ultrasonic imag* or ultrasonic tomograph* or ultrasonographic imag* or POCUS or "POC US" or "POC USG").tw,kw. | 46441 |
| 16 | 14 or 15 | 53932 |
| 17 | 13 and 16 | 68 |
| 18 | limit 17 to yr="2021 -Current" | 2 |
|  | | |

**Web of Science [Clarivate Analytics]**

**August 10 2022**

| **Set** | **Results** | **Searches** |
| --- | --- | --- |
| **11** | **35** | #9 AND #8 and 2022 or 2021 (Publication Years)  *Indexes=SCI-EXPANDED, SSCI, ESCI Timespan=All years* |
| **10** | **442** | #9 AND #8  *Indexes=SCI-EXPANDED, SSCI, ESCI Timespan=All years* |
| **9** | **522,295** | TS=(ultrasound* or ultrasonograph* or echograph* or echotomograph* or "medical sonography" or "ultrasonic diagnosis" or "ultrasonic diagnoses" or "ultrasonic imag*" or "ultrasonic tomograph*" or "ultrasonographic imag*" or POCUS or "POC US" or "POC USG")  *Indexes=SCI-EXPANDED, SSCI, ESCI Timespan=All years* |
| **8** | **31,245** | #7 OR #6 OR #5 OR #4 OR #3 OR #2 OR #1  *Indexes=SCI-EXPANDED, SSCI, ESCI Timespan=All years* |
| **7** | **1,380** | TS=(orbital NEAR/2 inflamm*)  *Indexes=SCI-EXPANDED, SSCI, ESCI Timespan=All years* |
| **6** | **280** | TS="Sinus venous thrombosis"  *Indexes=SCI-EXPANDED, SSCI, ESCI Timespan=All years* |
| **5** | **27,291** | TS=(sinusitis or rhinosinusitis) OR TS= (sinus NEAR/2 infect*)  *Indexes=SCI-EXPANDED, SSCI, ESCI Timespan=All years* |
| **4** | **1,147** | TS=(orbital NEAR/1 disease*)  *Indexes=SCI-EXPANDED, SSCI, ESCI Timespan=All years* |
| **3** | **54** | TS=(Bacterial NEAR/3 eye infect*)  *Indexes=SCI-EXPANDED, SSCI, ESCI Timespan=All years* |
| **2** | **897** | TS=((subperiosteal or "sub periosteal" or orbital or intraconal) NEAR/1 abscess*)  *Indexes=SCI-EXPANDED, SSCI, ESCI Timespan=All years* |
| **1** | **1,773** | TS=((periorbit* or orbit* or preseptal or "pre septal" or postseptal or "post septal") NEAR/3 celluliti*)  *Indexes=SCI-EXPANDED, SSCI, ESCI Timespan=All years* |

TS=((periorbit* or orbit* or preseptal or "pre septal" or postseptal or "post septal") NEAR/3 celluliti*)

TS=((subperiosteal or "sub periosteal" or orbital or intraconal) NEAR/1 abscess*)

TS=(Bacterial NEAR/3 eye infect*)

TS=(orbital NEAR/1 disease*)

TS=(sinusitis or rhinosinusitis) OR TS= (sinus NEAR/2 infect*)

TS="Sinus venous thrombosis"

TS=(orbital NEAR/2 inflamm*)
