# Supplementary material for: Diagnostic test accuracy of ultrasound for orbital cellulitis: A systematic review
Source: PLoS One. 2023 Jul 6;18(7):e0288011. doi: 10.1371/journal.pone.0288011 (PMC10325084; doi:10.1371/journal.pone.0288011)
Supplement: S3 Appendix — (DOCX) [file pone.0288011.s003.docx]

**S3 Appendix C: Data extraction template**

| **General** | **Reviewer 1** | **Reviewer 2** | **Consensus** |
| --- | --- | --- | --- |
| Study name |  |  |  |
| Title |  |  |  |
| Journal |  |  |  |
| Study Time Period |  |  |  |
| Study Design (e.g. cohort or case report) |  |  |  |
| Institution |  |  |  |
| Country of study |  |  |  |
| Contact Author |  |  |  |
| Funding |  |  |  |
| Conflict of Interest |  |  |  |
| **Population** |  |  |  |
| Age of patient |  |  |  |
| Gender |  |  |  |
| Setting (i.e. ED, hospital, clinic) |  |  |  |
| Describe clinical presentation |  |  |  |
| Etiology (i.e. organism grown, if relevant) |  |  |  |
| Diagnosed with orbital cellulitis? |  |  |  |
| Diagnosed with periorbital cellulitis? |  |  |  |
| Chandler Criteria (I, II, III, IV, V) |  |  |  |
| Patient outcome |  |  |  |
| **Imaging** |  |  |  |
| Ultrasound type (routine, POCUS) |  |  |  |
| Ultrasound mode, if relevant (B, M) |  |  |  |
| Describe US findings |  |  |  |
| Describe US findings of orbital cellulitis |  |  |  |
| Timing of US relative to initial presentation |  |  |  |
| Any other US details |  |  |  |
| CT scan type (head, neck, orbits, etc.) |  |  |  |
| With contrast? |  |  |  |
| Describe CT findings |  |  |  |
| Describe CT findings of orbital cellulitis |  |  |  |
| Timing of CT relative to initial presentation |  |  |  |
| Any other CT details |  |  |  |
| MRI scan type (head, neck, orbits, etc.) |  |  |  |
| With contrast? |  |  |  |
| Describe MRI findings |  |  |  |
| Describe MRI findings of orbital cellulitis |  |  |  |
| Timing of MRI relative to initial presentation |  |  |  |
| Any other MRI details |  |  |  |
