# Supplementary material for: Diagnostic test accuracy of ultrasound for orbital cellulitis: A systematic review
Source: PLoS One. 2023 Jul 6;18(7):e0288011. doi: 10.1371/journal.pone.0288011 (PMC10325084; doi:10.1371/journal.pone.0288011)
Supplement: S4 Appendix — (DOCX) [file pone.0288011.s004.docx]

**Appendix D: Characteristics of included cohort studies**

| **Study author, year** | **Participant characteristics** | **Presentation, prior testing** | **Clinical setting** | **Study design** | **Target condition definition** | **Index test** | **Reference standard** | **Sample size** | **Funding sources** |
| --- | --- | --- | --- | --- | --- | --- | --- | --- | --- |
| Goodwin, 1982 | All patients with a diagnosis of orbital cellulitis and/or abscess formation between July 1978 to June 1981 were reviewed | Patients presented with orbital edema, proptosis, and limitation of extra ocular motility. | University of Miami-Jackson Memorial Medical Center | Cohort study | No definition provided | Standardized orbital ultrasound (A-scan and B-scan) examinations were done and evaluated by experienced technicians | CT studies were done on a GE 8800 scanner and reviewed by neuroradiologist | 22 | Not reported |
| Kaplan, 1999 | sinus induced orbital infections | All patients presented with  pyrexia, chemosis, proptosis and limited eye movements  Edematous mucosa with purulent nasal discharge were observed by anterior rigid rhinoscopy in all patients | Soroka University Medical Center during an 18-month period | Cohort study | No definition provided | SOU was performed by an experienced ophthalmologist within 48 h of admission. | CT scan was performed within 24 h of hospital admission | 7 | Not reported |
| Mair, 2002 | swelling and erythema of the eyelids  patients ranged in age from 1 to 10 years |  | examined at the radiology unit of the Department of Pediatrics, Innsbruck University Hospital, | Cohort  study | No definition provided | Ultramark 8 HDI or HDI 5000 scanner equipped with a 5-8– MHz curved array transducer (C8-5) and a 5-12– MHz linear array ransducer (L12-5)  Orbital sonography was performed either immediately or at least within 12 hr after admission of the patients to the hospital. | Additional CT was performed in three patients. One was also examined with MR imaging; another patient was examined with MR imaging only | 17 | Not reported |

CT=Computed Tomography; MR=Magnetic Resonance
