# Supplementary material for: Diagnostic test accuracy of ultrasound for orbital cellulitis: A systematic review
Source: PLoS One. 2023 Jul 6;18(7):e0288011. doi: 10.1371/journal.pone.0288011 (PMC10325084; doi:10.1371/journal.pone.0288011)
Supplement: S5 Appendix — (DOCX) [file pone.0288011.s005.docx]

**S5 Appendix E: Table listing various ultrasound techniques**

| **Technique** | **Benefit** | **Reference** |
| --- | --- | --- |
| Moving the transducer as far as possible toward the lateral canthus of the eye. | Medial orbital wall can be viewed | Mair 2002 |
| Rocking the probe medially and laterally and focusing on the nasal and temporal tissues | Additional views could be obtained | Beam 2021; Seguin 2019 |
| Marker of the transducer can be oriented towards the right or head of the patient, respectively | To view transvers and longitudinal planes. | Seguin 2019; James 2018 |
| The transducer can also be fanned back and forth | For clear visibility of anterior and posterior of the eye | Seguin 2019 |
| The patient can be asked to move their eye or fix their gaze on objects | For assessing extraocular muscles | Seguin 2019 |
| Child can be distracted by a video on the parent’s smartphone | ocular POCUS could be performed | Seguin 2019 |
| Direct pressure or probe contact can be avoided by using a copious amount of ultrasound gel as an acoustic ‘‘stand-off’’ over the closed swollen eyelid covered with Tegaderm 3M transparent film | To prevent undue pressure and pain | James 2018 |
| The ocular ultrasound should be done in both sagittal and transverse planes | To assess the anterior segment, posterior segment, and post septal compartment of the eye | James 2018 |
| Both the optic disc height and optic nerve sheath diameter be measured. | They may indicate the development of concurrent intracranial complications like cavernous sinus thrombosis or brain abscess | James 2018 |
